# Supplementary material for: Development of germline progenitors in larval queen honeybee ovaries
Source: Biol Open. 2024 Sep 12;13(9):bio060511. doi: 10.1242/bio.060511 (PMC11413931; doi:10.1242/bio.060511)
Supplement: Supplementary information [file biolopen-13-060511-s1.pdf]

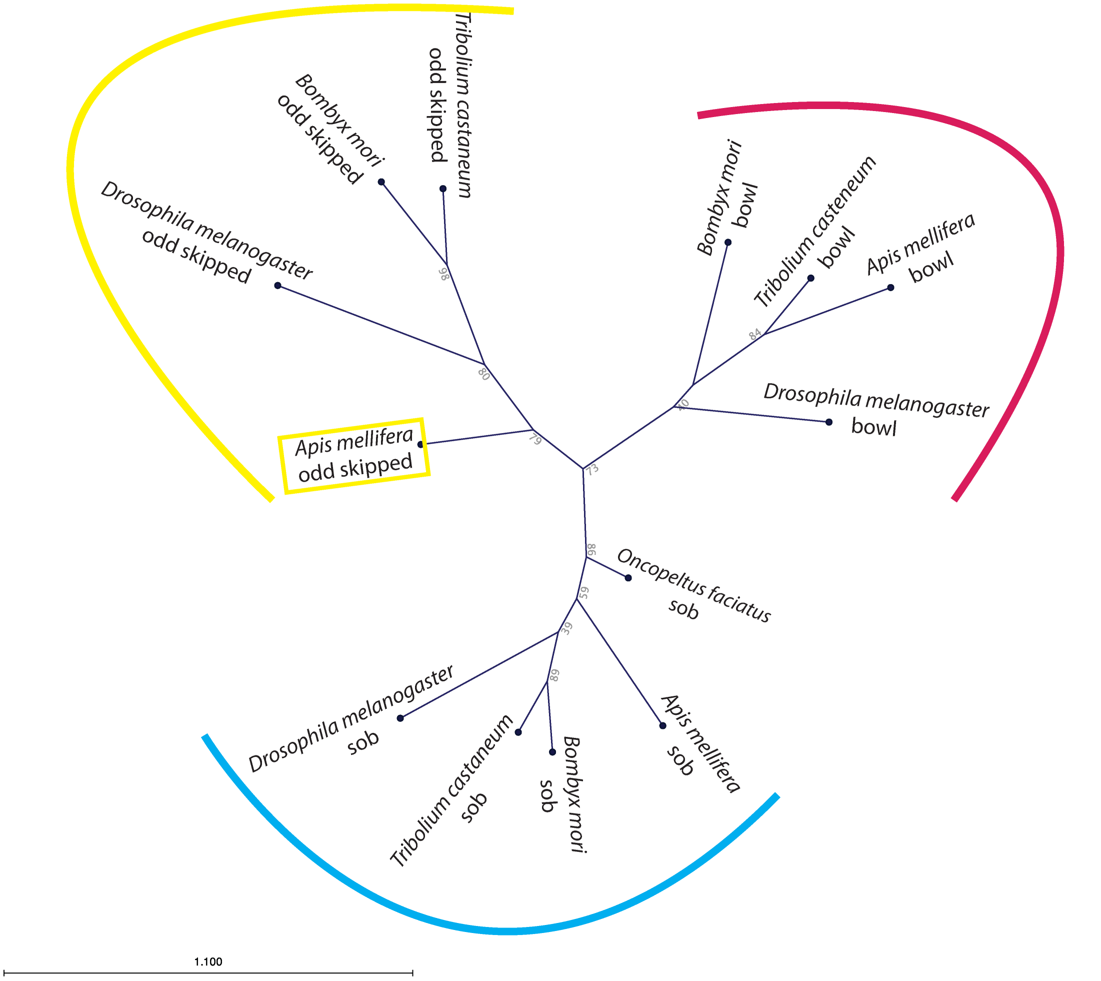

Supplemental Figure 1. Maximum-likelihood phylogram of representative insect odd skipped like proteins. *Apis mellifera* odd skipped (yellow box) is in a clade with odd skipped from other insects to the exclusion of sob and bowl proteins. Sequence identifiers are; *Drosophila melanogaster* odd, FBpp0077246; *Drosophila melanogaster* bowl, FBpp0077180; *Drosophila melanogaster* sob, FBpp0077247; *Apis mellifera* odd, XP_001120949; *Apis mellifera* sob, XP_026300496; *Apis mellifera* bowl, XP_026300446.1; *Tribolium castaneum* odd, XP_008196754.1; *Tribolium castaneum* sob, XP_008196753.1; *Tribolium castaneum* bowl, XP_008196755.1; *Bombyx mori* odd, XP_037877356.1; *Bombyx mori* sob, XP_012550570.3; *Bombyx mori* bowl, XP_004927088.3; *Oncopeltus faciatus* sob, AYR04656.1. Clade credibility values are bootstraps from 1000 replicates.

Fig. S1. Phylogenetics of odd skipped proteins from holometabolous insects

Biology Open • Supplementary information

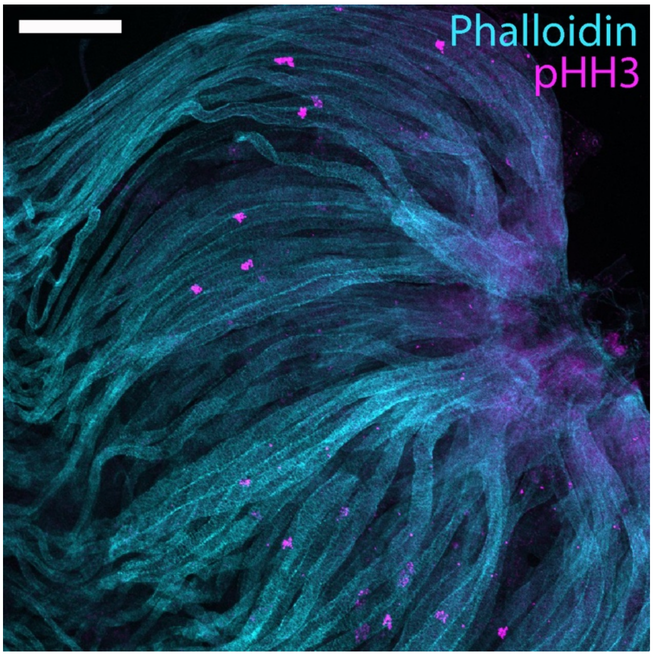

Fig. S2. Ovary of a newly emerged queen honeybee stained for pHH3 (magenta) nd cortical actin (cyan). Clusters of synchronously dividing cells can be seen in each ovariole.

Table S1. Staging of individual honeybees used in these experiments based on Cridge et al., (2017).

Stage	Days Post-Laying	Numbers of ovaries assayed across replicates
Larval Stage 2	5	17
Larval Stage 3	6	40
Larval Stage 4	7	30
Larval Stage 5 (Early)	8-9	27
Larval Stage 5 (Late)	10-11	31
Early Pupa	12-13	39
Late Pupa	15-16	28
Newly Emerged Queens	17+	15

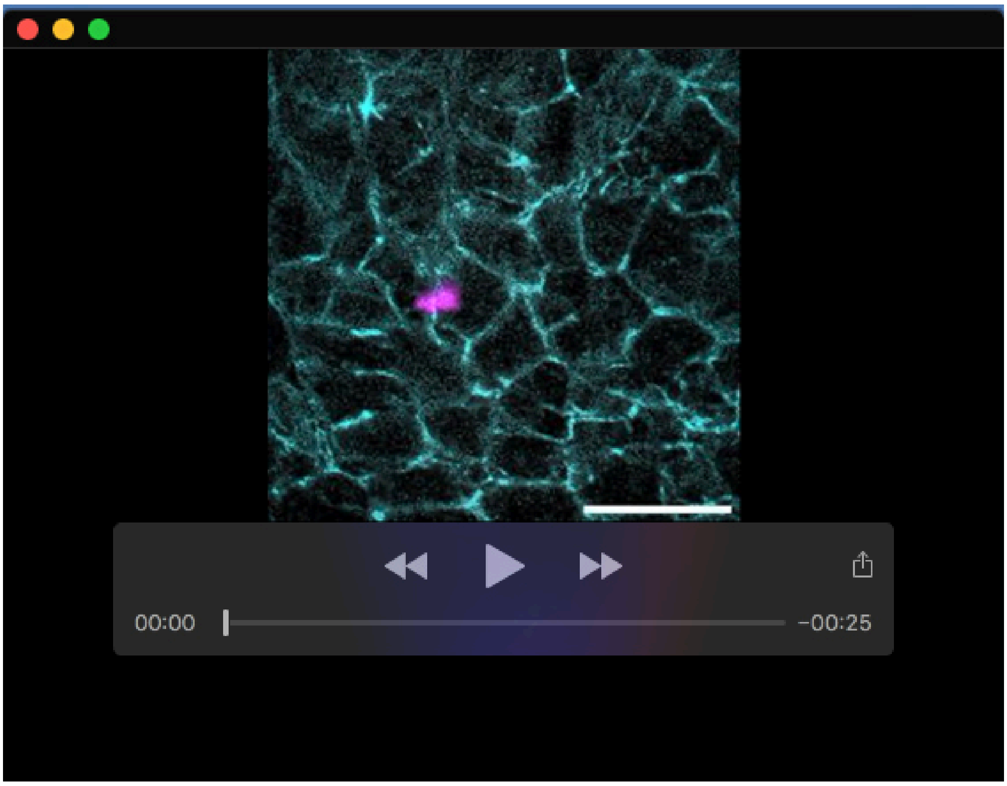

Movie 1. Progressive focus-through of a larval stage 3 ovary stained for phalloidin (cyan) and pHH3 (magenta) to detect dividing cells. The focus passes through a 4-cell cluster of cells dividing synchronously.

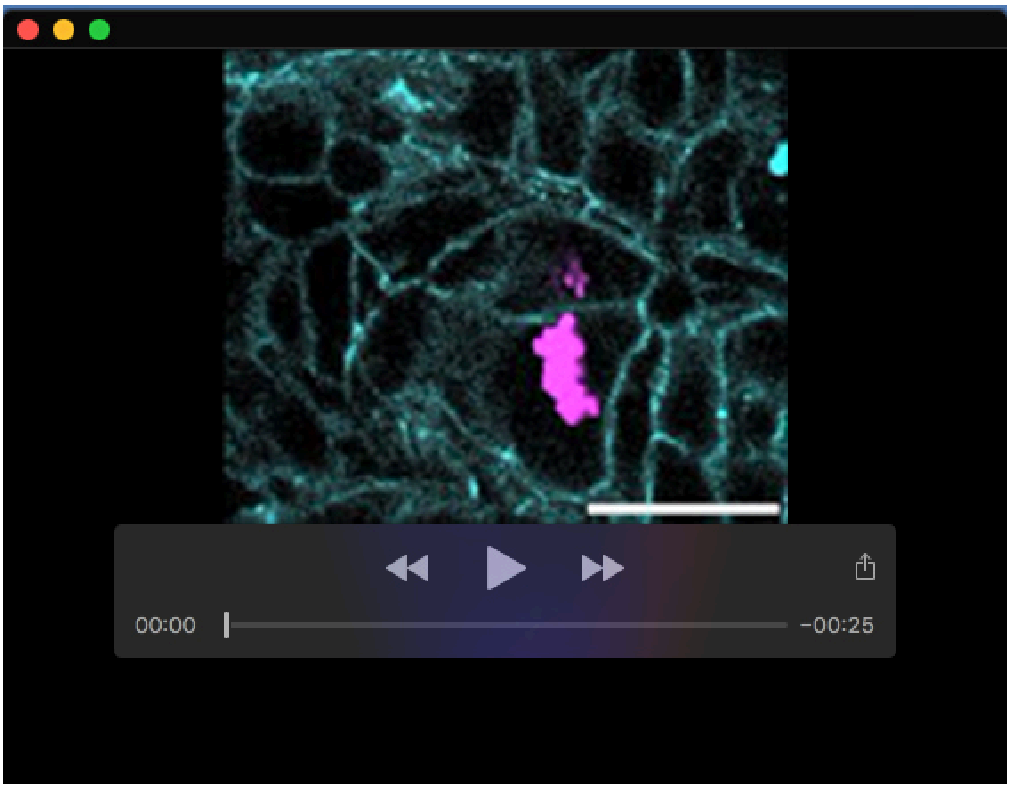

Movie 2. Progressive focus-through of a larval stage 4 ovary stained for phalloidin (cyan) and pHH3 (magenta) to detect dividing cells. The focus passes through a 4-cell cluster of cells dividing synchronously.

Biology Open • Supplementary information

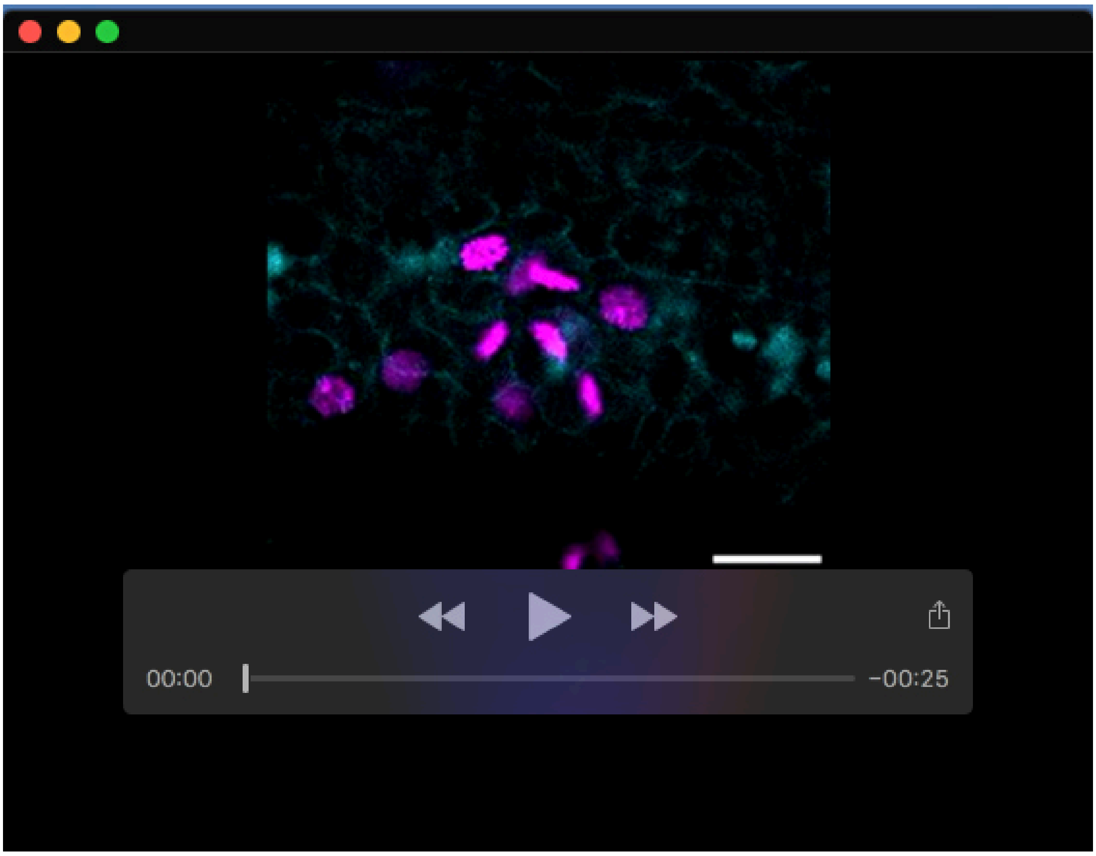

Movie 3. Progressive focus-through of a larval stage 5 ovary stained for phalloidin (cyan) and pHH3 (magenta) to detect dividing cells. The focus passes through a 8-cell cluster of cells dividing synchronously.

Biology Open • Supplementary information
